# MACNeXt-Based Bacteria Species Detection

**DOI:** 10.3390/microorganisms13122689

**Published:** 2025-11-25

**Authors:** Ozlem Aytac, Feray Ferda Senol, Tarik Kivrak, Zulal Asci Toraman, Mehmet Veysel Gun, Omer Faruk Goktas, Sengul Dogan, Turker Tuncer

**Affiliations:** 1 Elazig Fethi Sekin City Hospital, Medical Microbiology, 23200 Elazig, Türkiye; ozlemozlem5@hotmail.com (O.A.); drferdasenol@yahoo.com (F.F.S.); 2 Department of Cardiology, Faculty of Medicine, Fırat University, 23119 Elazig, Türkiye; 3 Department of Microbiology, Faculty of Medicine, Fırat University, 23119 Elazig, Türkiye; zulalasci@gmail.com; 4 Department of Digital Forensics Engineering, Technology Faculty, Firat University, 23119 Elazig, Türkiye; mvgun@firat.edu.tr (M.V.G.); sdogan@firat.edu.tr (S.D.); turkertuncer@firat.edu.tr (T.T.); 5 Department of Electronics and Automation, Technical Sciences Vocational School, Ankara Yildirim Beyazit University, 06010 Ankara, Türkiye; ofgoktas@aybu.edu.tr

**Keywords:** bacterial identification, microbial image analysis, deep learning, CNN, biomedical image classification

## Abstract

Bacteria underpin human health, environmental balance, and industrial processes. Rapid and accurate identification is essential for diagnosis and responsible antibiotic use. Culture, biochemical tests, and microscopy are slow, expensive, and depend on expert judgment, which introduces subjectivity and errors. This research aims to recommend a new generation deep learning architecture for bacterial species classification. We curated a bacterial image dataset, and this dataset contains 18,221 microscopic images from 24 species under standard laboratory conditions. All images passed clarity and focus checks. We developed a compact CNN, the Multiple Activation Network (MACNeXt). The recommended MACNeXt preserves local feature extraction and improves representation with two activation functions (GELU and ReLU) and a multi-branch design. The aim is high accuracy with low computational cost for routine clinical use. MACNeXt achieved 90.97% accuracy, 89.63% precision, 88.64% recall, and 88.99% F1-score on the test set. The calculated results and findings showcase balanced and stable performance across species with an efficient, lightweight design since the introduced MACNeXt has about 4.4 million learnable parameters. The results of the MACNeXt openly demonstrate that this CNN is a compact, lightweight, and highly accurate CNN model.

## 1. Introduction

Bacteria are essential to human health, environmental balance, and industrial systems [1,2]. Accurate detection and classification are critical for the diagnosis and treatment of infectious diseases [3]. Traditional identification relies on culture growth, biochemical reactions, and microscopic observation. These procedures are slow, costly, and require expert judgment, which limits rapid diagnostics. Human interpretation also adds subjectivity and increases error risk [4,5].

Advances in artificial intelligence now support deep learning for microscopic image analysis [6]. Deep models identify complex visual patterns in large datasets with higher speed, consistency, and accuracy than manual techniques. This capability lowers human error and improves clinical workflow efficiency [7].

Deep learning models/architectures, especially Convolutional Neural Networks (CNNs), showcase strong performance in microbiological image classification through effective visual feature extraction [8].

In this research, a CNN model was built on a custom bacterial image dataset. We curated a large and diverse dataset, and this dataset contains 18,221 bacterial images with 24 classes. Performance evaluation covered accuracy, F1-score, and the confusion matrix, and results were compared with prior work. The outcomes indicate that deep learning can strengthen bacterial identification systems for both research and clinical use.

Li et al. [9] introduced a TFT-based lens-free imaging system for early bacterial colony detection. Their dataset included 265 colonies from Escherichia coli, Citrobacter, and Klebsiella pneumoniae. Colonies were imaged every five minutes, and a ResNet-based deep model was trained on these sequences. The system achieved 97.3% colony-forming unit (CFU) accuracy and a 91.6% recovery rate after nine hours. It produced results about 12 h earlier than traditional microbiology. They used a limited number of bacterial classes and a small dataset; they used a well-known ResNet architecture without major architectural innovation. Yang et al. [10] applied style transfer–based augmentation and deep learning for bacterial colony detection on the AGAR dataset. About 4000 synthetic samples were generated to address data scarcity. Cascade Mask R-CNN + Swin Transformer and YOLOv8x models were trained, reaching 76.7% and 61.4% detection accuracy, respectively. The main limitation of their work is given as follows: limited dataset diversity and dependency on artificial samples, where models relied on existing architectures with no new structural design. Wu and Gadsden [11] compared well-known pre-trained CNNs, including DenseNet-121, ResNet-50, and VGG16, on 660 microbial images from 33 bacterial species. DenseNet-121 achieved the best results with 99.08% accuracy and 98.99% F1-score. They used a small dataset and focused only on pretrained CNNs, lacking novelty in architecture or data expansion. Makrai et al. [12] created a large dataset of 56,865 colonies from 24 veterinary bacterial species. Colonies on 369 Petri dishes were annotated with bounding boxes, and the dataset was made publicly available to support AI-based colony counting and classification research. The work focused on dataset creation without proposing a new model or classification framework. Talo [13] presented a transfer learning approach using a pre-trained ResNet-50 model for 33 bacterial species from the DIBaS dataset. The model achieved 99.2% accuracy through fine-tuning. This research relied fully on an existing model and dataset; it lacked architectural novelty and limited the analysis to a fixed dataset. Akbar et al. [14] recommended a hybrid model combining ResNet-101 feature extraction and SVM classification. The method achieved 99.61% accuracy, 99.58% precision, and 99.58% recall on 660 images. They utilized a very small dataset and a well-known hybrid structure; no innovation in feature extraction or learning strategy. Gallardo-García et al. [15] tested lightweight CNNs such as EfficientNet-Lite0 and MobileNetV2 on the DIBaS dataset. Artificial zoom–based augmentation improved results, achieving 97.38% accuracy. They applied the well-known models to an available dataset. Therefore, this research aims to analyze deep learning for bacteria classification. Mai and Ishibashi [16] developed a depthwise separable CNN for efficient bacterial classification on the DIBaS dataset. The model maintained low computational cost with high accuracy. Their model is a VGG-like model. Thus, their model’s innovation is low. Zieliński et al. [17] recommended a CNN-based classifier using 660 DIBaS images from 33 species, achieving 97.24% accuracy. This dataset is a relatively small dataset. Hallström et al. [18] used microfluidic chip–based phase-contrast imaging to classify four bacterial species. A video-based ResNet trained on 27-frame sequences achieved 99.55 ± 0.25% accuracy. They used ResNet-based model to guarantee high classification performance. Kang et al. [19] combined hyperspectral microscopy (HMI) and CNNs for single-cell bacterial classification. The 1D-CNN achieved 90% accuracy. The single-cell dataset had a narrow scope; it had limited innovation in model structure and low overall accuracy compared with image-based models. Abd Elaziz et al. [20] proposed a hybrid model combining fractional-order orthogonal descriptors and semantic feature extraction on the DIBaS dataset, achieving 98.68% accuracy. They used a known public dataset and hybridized traditional descriptors with deep learning, with no architectural novelty and limited generalization to real-world data.

### 1.1. Literature Gaps

Most previous studies used small or limited datasets. The present work employed a large and diverse dataset to improve model reliability and generalization.

Many studies failed to address the challenge of separating morphologically similar bacterial species such as Staphylococcus and Streptococcus. Solving this issue requires new architectures and advanced feature extraction strategies.

No comprehensive research exists on integrating AI-based bacterial identification systems into real clinical workflows. User experience and clinical acceptance have also not been systematically investigated.

### 1.2. Motivation and Our Model

Accurate bacterial species classification directs diagnosis and antibiotic choice [5]. Conventional culture, Gram stain, and biochemical assays run slowly [21]. Reports often require 24–72 h and expert review. Delays risk patient safety, especially in emergency care. Subjective evaluation yields variability and diagnostic errors across sites [22].

Advances in deep learning enable objective image-based identification [23]. The deep learning models and the advanced image processing models capture subtle visual cues beyond human perception [24]. Systems operate continuously and sustain consistent decisions. These properties address speed, standardization, and reliability [25].

This study responds with a CNN-based classifier. We assembled 18,221 microscopic images from 24 clinical species under standard protocols. The dataset reflects real laboratory variance and supports fair evaluation and robust training.

The recommended deep learning model, MACNeXt, derives from ConvNeXt and remains fully convolutional. Dual activations (GELU, ReLU) expand nonlinear capacity. A multi-branch design and a two-layer squeeze unit refine features. Grouped 3 × 3 stride-2 layers halve spatial size and double depth. The network uses 4.4M parameters. Test accuracy reaches 90.97% with AUC 0.9499.

Balanced metrics confirm robustness: precision is 89.63%, recall is 88.64%, and F1 is 88.99%. The compact design fits laboratories with limited hardware and supports fast, standardized, reproducible practice.

### 1.3. Novelties

A large and diverse dataset was collected for this research, and this dataset has 18,221 images with 24 classes.

An innovative CNN model has been recommended in this research, and this CNN is termed MACNeXt.

### 1.4. Contributions

By proposing MACNeXt and collecting a new bacterial image dataset, this research contributes to both new generation accurate CNN model proposing and bacterial image classification research areas.

## 2. Materials and Methods

### 2.1. Materials

This study followed the standard procedures of the Department of Medical Microbiology, Elazig Fethi Sekin City Hospital. Data came from routine diagnostic cultures that showed bacterial growth. Specimen types included urine, tracheal aspirate, sputum, wound swab, blood, cerebrospinal fluid, vaginal secretion, semen, and other sterile body fluids. Each specimen was incubated on 5% sheep blood agar for 24–48 h to observe growth. Identification relied on conventional microbiology and the automated Vitek 2.0 system.

After identification, each colony on the agar plate was photographed separately. Images were acquired with a Canon EOS 70D digital camera fitted with an 18–55 mm lens. Illumination was controlled to maintain image clarity, correct focus, and uniform exposure across all samples.

**Figure 1:**
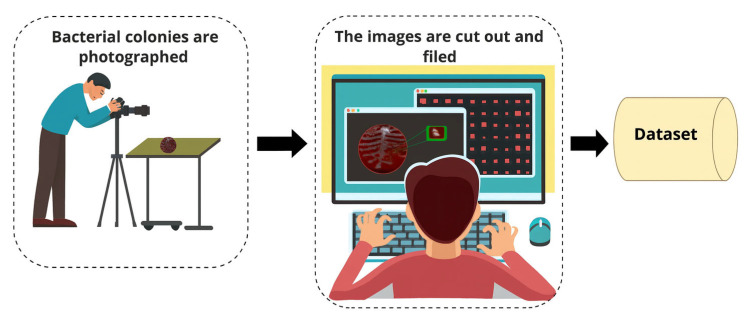
Workflow of bacterial colony image acquisition and dataset preparation.

Figure 1 showcases agar plate colonies photographed under controlled laboratory conditions with a Canon EOS 70D. The images were moved to a computer system. Each colony was cropped, organized, and labeled by species. The result was a structured image dataset suitable for later analysis.

The dataset covered 24 bacterial species across 24 classes. Table 1 lists each class with the species name, sample count, and one representative image.

### 2.2. The Proposed MACNeXt

This research presents a next-generation CNN, Multiple Activation Network (MACNeXt). The recommended MACNeXt model is lightweight and targets both classic CNN design and efficient deployment. We also prepared a bacterial image dataset with 24 classes, each mapped to a distinct species. The introduced MACNeXt is inspired by the presented ConvNeXt. It reduces the number of normalization layers to improve efficiency. Inspired by the Kolmogorov–Arnold Network (KAN) idea, the network uses two activations: GELU and ReLU.

The stem block has two 3 × 3 convolutions that form the first tensor. The core block departs from conventional CNNs with concatenation and dual activations, which yield richer feature maps. Residual links preserve information flow and stabilize gradients. A two-layer squeeze unit and a multi-branch layout provide channel-wise emphasis akin to attention.

**Figure 2:**
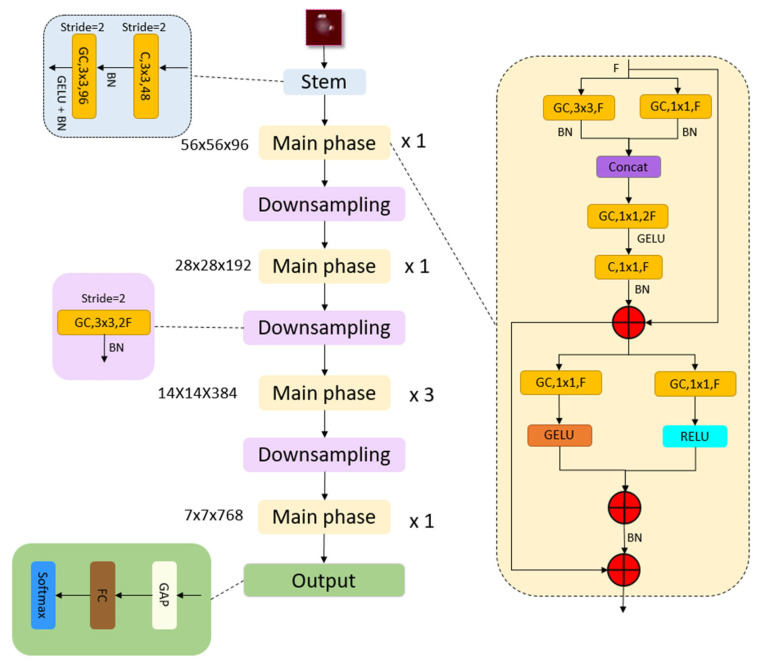
The overall architecture of the proposed MACNeXt model, illustrating the stem, main, and downsampling phases. Herein, C(.): convolution operation, GC(.): grouped convolution operation, Concat(.): depth concatenation, BN(.): Batch Normalization, GAP(.).

The downsampling stage uses 3 × 3 grouped convolutions (stride 2) to halve spatial size and double channel depth. In the output layer, a straightforward output phase has been utilized to show the high feature map generation ability of the recommended MACNeXt. The architecture of the proposed MACNeXt model is shown in Figure 2.

Stem Block: The model begins with an input image of size 224 × 224 × 3 in RGB format. The first layer is a 3 × 3 convolution with 48 filters and a stride of 2, followed by Batch Normalization (BN). Next, a second 3 × 3 grouped convolution with 96 filters and the same stride is applied, supported by BN and GELU activation. These operations produce a 56 × 56 × 96 feature map.

The mathematical representation of this block is given below.

Apply the recommended stem block to generate the first tensor.

(1) $${Tens}_{1}=BN(G\left({GC}_{96,S:2}^{3\times 3}\left(BN\left(C_{48,S:2}^{3\times 3}\left(Images\right)\right)\right)\right))$$

Herein, ${Tens}_{1}$: the first tensor, C(.): convolution operation,$\;BN\left(.\right):$ Batch Normalization, $G\left(.\right):$ GeLU activation function, and GC(.): grouped convolution operation.

Main block: The main block functions as the core refinement unit that processes the F-channel input at multiple scales and reorganizes it through stable residual connections. Parallel 3 × 3 and 1 × 1 convolution paths extract local textures and cross-channel information. Their outputs are merged using concatenation, expanding the channel dimension to 2F. A subsequent 1 × 1 convolution reduces the width back to F, forming an expand–squeeze bottleneck structure.

Two middle branches apply GELU and ReLU activations to model both smooth and sparse nonlinear behaviors. Multi-level residual summation nodes maintain information flow and improve gradient stability. Batch Normalization (BN) ensures consistent data distribution and stable training. A long skip connection at the bottom links the block input to the final output, reducing information loss and enhancing feature retention.

The step-by-step mathematical form of this block is given below.

Apply one 3 × 3 and one 1 × 1 grouped convolution on the input tensor ${Tens}_{n-1}$. Normalize both outputs with Batch Normalization (BN) and merge them along the channel axis. Capture spatial context with the 3 × 3 path and channel mixing with the 1 × 1 path simultaneously to produce a richer feature map.

(2) $${Tens}_{n}=Concat\left(BN\left({GC}_{F}^{3\times 3}\left({Tens}_{n-1}\right)\right),BN\left({GC}_{F}^{1\times 1}\left({Tens}_{n-1}\right)\right)\right)$$

Herein, ${Tens}_{n}$: nth tensor, ${Tens}_{n-1}:$ previous tensor, and Concat(.): the depth concatenation function. We have used depth concatenation to increase the number of filters for creating an inverted bottleneck.

Project the concatenated tensor to 2F channels using a 1 × 1 grouped convolution.

(3) $${Tens}_{n+1}={GC}_{2F}^{1\times 1}\left({Tens}_{n}\right)$$

Pass the expanded tensor through GELU, then reduce it back to F channels using a 1 × 1 convolution, followed by Batch Normalization (BN).

(4) $${Tens}_{n+2}=BN\left(C_{F}^{1\times 1}\left(G\left({Tens}_{n+1}\right)\right)\right)$$

Perform the first residual addition (skip connection):

(5) $${Tens}_{n+3}={Tens}_{n-1}+{Tens}_{n+2}$$

Apply two parallel GELU and RELU activations to the same input.

(6) $${Tens}_{n+4}=G\left(GC_{F}^{1\times 1}\left({Tens}_{n+3}\right)\right)$$

(7) $${Tens}_{n+5}=R\left({GC}_{F}^{1\times 1}\left({Tens}_{n+3}\right)\right)$$

Herein, $R\left(.\right):$ ReLU activation function.

Apply addition to the GELU and ReLU branches, then apply Batch Normalization (BN).

(8) $${Tens}_{n+6}=BN({Tens}_{n+4}+{Tens}_{n+5})$$

Apply the shortcut to the previous intermediate tensor directly to the fused features.

(9) $${Tens}_{out}={{Tens}_{n+3}+Tens}_{n+6}$$

Downsampling: Each downsampling layer applies a 3 × 3 grouped convolution with a stride of 2, followed by Batch Normalization (BN), to reduce the spatial dimensions by half. The feature map sizes change progressively as follows: 56 × 56 × 96 → 28 × 28 × 192 → 14 × 14 × 384 → 7 × 7 × 768.

This hierarchical process allows the model to extract deeper and more abstract representations at each level. The output of the previous block is adjusted through a learnable downsampling step to ensure a smooth transition and compatibility with the next stage of the network.

Apply a 3 × 3 grouped convolution with stride 2 to reduce spatial resolution by half while doubling the channel count from F to 2F.

Apply Batch Normalization (BN) to balance distributions and ensure stable training.

(10) $${Tens}_{ds}=BN\left(GC_{2F,\;S:2}^{3\times 3}\left({Tens}_{out}\right)\right)$$

Herein, ${Tens}_{ds}$: the downsampled tensor.

Output: The GAP layer summarizes spatial information and prevents overfitting by removing location bias.

The FC layer maps condensed features to target class dimensions.

Softmax converts these scores into normalized probabilities, ensuring all class values sum to one.

This structure produces a compact and interpretable output suitable for multi-class prediction.

(11) $$out=softmax\left(FC\left(GAP\left({Tens}_{final}\right)\right)\right)$$

where out: the classification outcome, FC(.): fully connected layer, GAP(.): global average pooling, and $Tens_{final}$: the last generated tensor with a size of 7 × 7 × 768.

The transition table of the recommended MACNeXt is also demonstrated in Table 2.

To better explain the recommended MACNeXt architecture, the pseudocode of this model is showcased in Algorithm 1.
**Algorithm 1.** Pseudocode of the introduced MACNeXt CNN architecture**Input:** RGB image I of size 224 × 224 × 3 **Output:** Class probabilities y_hat# ---------- Stem Block ----------
# Conv 3 × 3, 48 filters, stride 2 T1 = Conv 3 × 3(I, filters = 48, stride = 2)
T1 = BatchNorm(T1) # Grouped Conv 3 × 3, 96 filters, stride 2 + GELU
T1 = GroupConv 3 × 3(T1, filters = 96, stride = 2) T1 = GELU(T1)
T1 = BatchNorm(T1) # T1: 56 × 56 × 96 (Tens1)
# ---------- Stage configuration ----------
# Feature sizes per stage
F = [96, 192, 384, 768]
# Block repeat numbers per stage R = [1, 1, 3, 1]
T = T1
# ---------- Main Stages + Downsampling ----------
**for** s in 1...4 **do**                # stage index
        # ----- Repeat MACNeXt main block R[s] times -----
        **for** r in 1..R[s] **do**
                T = MACNeXt_Block(T, F[s])
        **end for**
        # ----- Apply downsampling after first 3 stages -----
        **if** s < 4 **then**
                # Grouped Conv 3 × 3, stride 2, channels: F[s] → 2·F[s]
                T = GroupConv 3 × 3(T, filters = 2 × F[s], stride = 2)
                T = BatchNorm(T)
                # now channel size becomes F[s + 1]
        **end if** **end for** # ---------- Classification Head ----------
T_gap = GlobalAveragePooling(T)              # 1 × 1 × 768
z         = FullyConnected(T_gap, n)         # logits, Herein, n is the number of outputs
y_hat = Softmax(z)                                        # probabilities
return y_hat
# ---------- Definition of MACNeXt main block ----------
# Input: tensor X with F channels # Output: tensor Y with F channels
**function MACNeXt_Block**(X, F):
        # (1) Parallel 3 × 3 and 1 × 1 grouped convolutions + BN
        P1 = GroupConv 3 × 3(X, filters = F, stride = 1)
        P1 = BatchNorm(P1)
        P2 = GroupConv 1 × 1(X, filters = F)
        P2 = BatchNorm(P2)
        # (2) Concatenate and expand (inverted bottleneck)
        C = ConcatChannels(P1, P2)                   # 2F channels
        E = GroupConv 1 × 1(C, filters = 2 × F)      # expansion
        # (3) GELU + squeeze to F channels + BN
        E = GELU(E)
        S = Conv 1 × 1(E, filters = F)
        S = BatchNorm(S)
        # (4) First residual connection
        R1 = X + S
        # (5) Dual activations on R1
        G_branch = GELU(GroupConv 1 × 1(R1, filters = F))
        R_branch = ReLU(GroupConv 1 × 1(R1, filters = F))
        # (6) Fuse branches + BN
        Fused = G_branch + R_branch
        Fused = BatchNorm(Fused)
        # (7) Second residual connection
        Y = R1 + Fused        return Y
**end function**

MACNeXt is inspired by ConvNeXt but shaped into a lighter and more practical form. The stem block converts the input image into a 56 × 56 × 96 tensor using two simple convolutions. The main block refines this tensor with an inverted bottleneck and dual activations. Parallel 3 × 3 and 1 × 1 grouped convolutions capture local textures and cross-channel patterns. Their fusion creates richer features without increasing the model size.

GELU and ReLU work together inside the block. GELU models smooth and low-energy signals, while ReLU increases sharp and sparse structures. Residual paths keep gradients stable and help the network preserve useful information. Downsampling stages reduce spatial size step by step and increase channel depth. After these stages, global average pooling and a small fully connected layer map the final tensor to n output classes.

The introduced MACNeXt has only 4.4 million parameters.

## 3. Performance Evaluation

In this research, a new-generation CNN model named MACNeXt was proposed. The model was applied to the curated bacterial image dataset. The default configuration of MATLAB 2023a was used for all experiments.

The network was implemented in MATLAB R2023a using the Deep Network Designer interface. All training parameters and their detailed descriptions are listed in Table 3. Experiments were carried out on a workstation equipped with a 14th Gen Intel Core i7 CPU, an NVIDIA GeForce RTX 4070 GPU, and 64 GB RAM.

**Figure 3:**
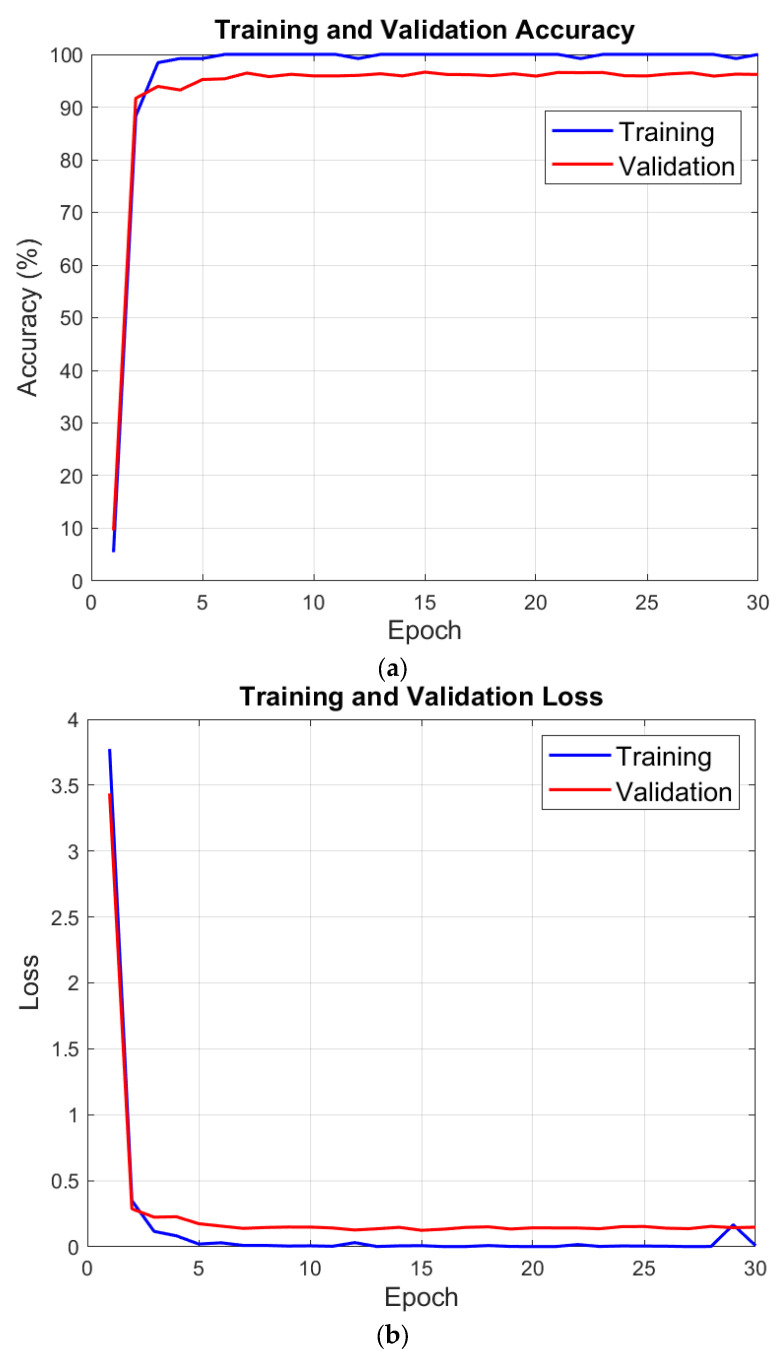
Performance variations observed during the model training process: (**a**) Change in training and validation accuracy across epochs. (**b**) Change in training and validation loss across epochs.

Table 3 reports the split protocol. We first allocated 80% of the data to training and 20% to testing. Deep Network Designer then partitioned the training set into 80% train and 20% validation, yielding 64%/16%/20% for train/validation/test. We recorded accuracy and loss for training and validation after each epoch to assess learning progress, generalization, and convergence. Figure 3 displays these results.

Validation is 97.46% at the end of the training phase with MACNeXt. We then evaluated the test set with the pretrained model. A confusion matrix measures test performance by comparing predicted and true labels. Diagonal cells indicate correct predictions; off-diagonal cells indicate misclassifications. This layout allows detailed analysis of overall accuracy and class-level performance.

**Figure 4:**
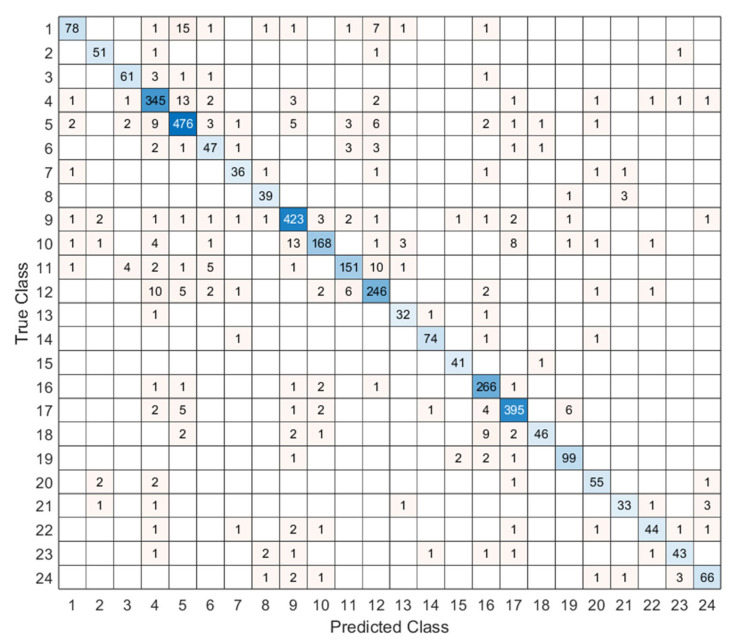
Confusion matrix of the proposed MACNeXt model on the test dataset, showing the classification performance across 24 bacterial species.

Figure 4 showcases the confusion matrix for 24 classes. Each cell count predicts labels for a given true label. A dense diagonal signals high accuracy. Top performance appears in classes 3, 5, 9, 16, and 17. Confusions cluster between 1 and 3 and between 10 and 11, likely due to morphological overlap and mild imbalance. Future work will add class-specific augmentation and stronger feature extractors to improve separation. We use port accuracy, precision, recall, and F1-score to evaluate the test results, and to compute these metrics, the given confusion matrix in Figure 4 is utilized. To compute these classification assessment metrics, we use true positives (TPs), false positives (FPs), true negatives (TNs), and false negatives (FNs) for each class. The mathematical explanations of these assessment metrics have been illustrated below.

(12) $$Accuracy=\frac{TP+TN}{TP+TN+FP+FN}$$

(13) $$Precision=\frac{TP}{TP+FP}$$

(14) $$Recall=\frac{TP}{TP+FN}$$

(15) $$F1-Score=2\frac{Precision\times Recall}{Precision+Recall}$$

**Figure 5:**
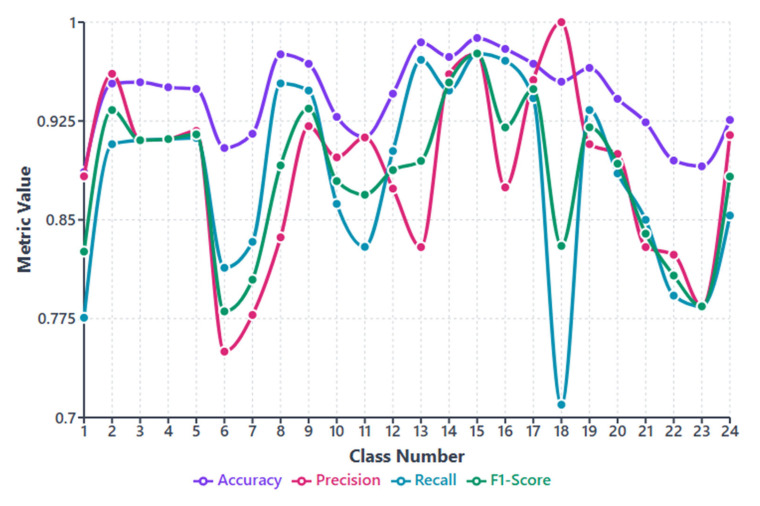
Per-class performance metrics (accuracy, precision, recall, and F1-score) for the proposed MACNeXt model across 24 bacterial species.

Table 4 reports accuracy, precision, recall, and F1-score. All metrics exceed 88%, which reflects stable and consistent performance. Overall accuracy is 90.97%. MACNeXt yields reliable classification. The results support a balanced design with strong overall accuracy and correct identification of positive samples. For deeper insight, we examined metrics per class. The class-wise performance evaluation is also demonstrated in Figure 5.

Figure 5 showcases accuracy, precision, recall, and F1-score for each class. This figure (see Figure 5) helps us see how class imbalance affects the model. The dataset has large differences between classes. For example, class 5 includes 2562 samples, while class 13 has only 177. This uneven (imbalanced) distribution creates performance changes across species.

Most classes show stable results above 0.85 in all metrics. These classes either have clearer colony shapes or enough samples for strong learning. Classes 3, 5, 9, 16, and 17 performed the best and remained the most consistent.

Some smaller classes show slight drops. Class 6, class 13, and class 18 have lower recall and F1-scores. These classes have limited data or look similar to other species. Class 18 shows the biggest recall drop because its colony patterns overlap with other Staphylococcus groups. This suggests that limited training examples make these classes harder to separate.

Even without class weighting, MACNeXt handles imbalance well. The dual activations (GELU + ReLU) and the multi-branch design help the model capture detailed textures. These features reduce performance loss from imbalance, although very small classes remain more challenging.

## 4. Discussion

MACNeXt reached balanced results through architectural symmetry and controlled optimization, not through class weights. Dual activations (GELU + ReLU) improved gradient flow in low- and high-activation regimes; GELU stabilized small signals, ReLU enforced sparsity, which limited majority-class dominance. The multi-branch paths (parallel 3 × 3 and 1 × 1) extracted fine textures and coarse colony structure, so minority classes gained distinct features without parameter bloat. The two-layer squeeze unit recalibrated channels with data-driven weights and raised the influence of minority-discriminative filters. Grouped convolutions reduced inter-channel correlation and equalized gradient norms across blocks. Batch composition used class-aware mini-batches (size 128) so each batch preserved a near-uniform class mix. Optimization used SGDM (learning rate = 0.01) with Batch Normalization, which stabilized the loss landscape under standard cross-entropy and prevented drift toward head classes. Under this setup, macro and micro metrics stayed close, and per-class precision, recall, and F1 remained ≥ 0.88 despite label imbalance.

Misclassifications mostly happen in species that look very similar. The clearest example is between the Staphylococcus and Streptococcus groups. Their colonies often share close shapes, colors, and edge patterns on blood agar. Because of this similarity, the model can confuse these classes, especially when sample numbers are low or the colony borders are not very clear.

A closer look at the confusion matrix shows that most errors appear in borderline colonies where the morphology is difficult to distinguish. These cases are also challenging for human experts. Increasing the number of images for these species or collecting samples under different lighting and imaging conditions may reduce these errors in future studies.

### 4.1. Comparative Results

**Figure 6:**
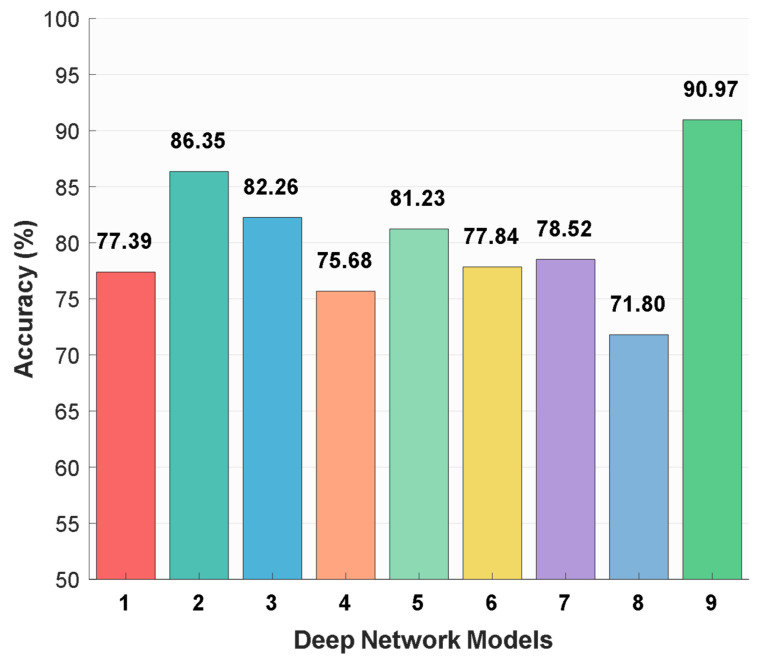
Classification accuracy rates (%) of different deep learning models on the curated dataset: 1: MobileNetV2 [26], 2: ResNet-50 [27], 3: DarkNet-53 [28], 4: AlexNet [29], 5: DenseNet-201 [30], 6: Inception-v3 [31], 7: Inception-ResNet-v2 [32], 8: GoogLeNet [33], and 9: MACNeXt (proposed).

MACNeXt was compared with the well-known CNN models to evaluate its position among existing architectures. Eight benchmark models from the literature were included: MobileNetV2, ResNet-50, DarkNet-53, AlexNet, DenseNet-201, Inception-v3, Inception-ResNet-v2, and GoogLeNet. Each model was trained and tested on the same dataset under the same conditions. The comparison used classification accuracy (%) to provide a fair and direct performance evaluation. These comparative results are shown in Figure 6.

The utilized DIBaS dataset includes a direct comparison between MACNeXt and the widely used CNN architectures. As showcased in Figure 6, baseline accuracies on the curated dataset vary between 71.80% and 86.35%. GoogLeNet attains the lowest result (71.80%), while ResNet-50 reaches the highest baseline value (86.35%).

**Figure 7:**
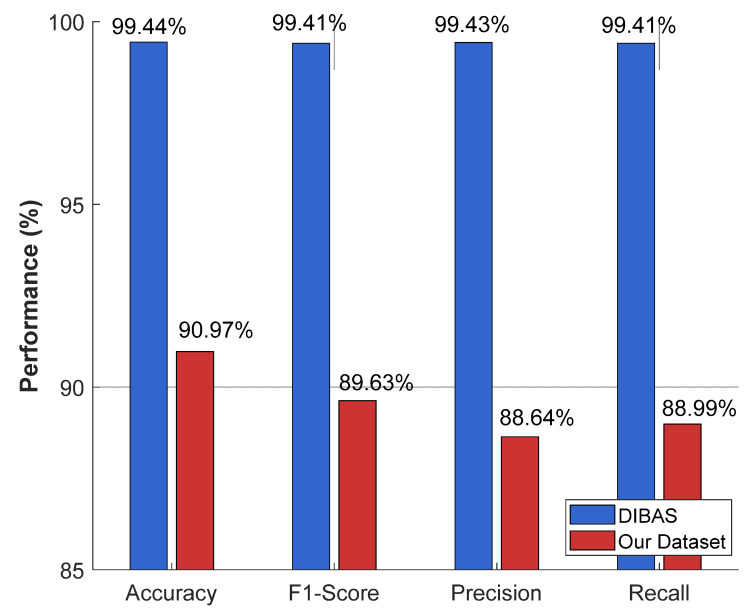
Comparative performance of the proposed MACNeXt model on the newly curated bacterial dataset and the DIBaS dataset. The DIBaS results were obtained from prior benchmark studies, whereas the “Our Dataset” results represent the outcomes achieved in this study.

The recommended MACNeXt model was tested on the publicly available DIBaS dataset. All models were trained under the same settings and preprocessing steps to provide a fair comparison. The recommended MACNeXt achieves over 99% test results. The comparative results of the recommended MACNeXt for our curated dataset and DIBaS dataset are showcased in Figure 7.

Figure 7 illustrates that the proposed MACNeXt achieved over 99% test classification performance on the DIBaS. Real-world samples have higher intra-class variation and more noise, which explains this gap. To showcase the high classification performance of the presented MACNeXt, we compared our results to other models. We have used DIBaS to obtain comparative results, and these comparative results are tabulated in Table 5.

Moreover, the recommended model has been tested on the blood cell image dataset, and we applied the recommended MACNeXt to blood cell images using the same training and validation hyperparameters, and the computed training and validation results are given in Table 6.

**Figure 8:**
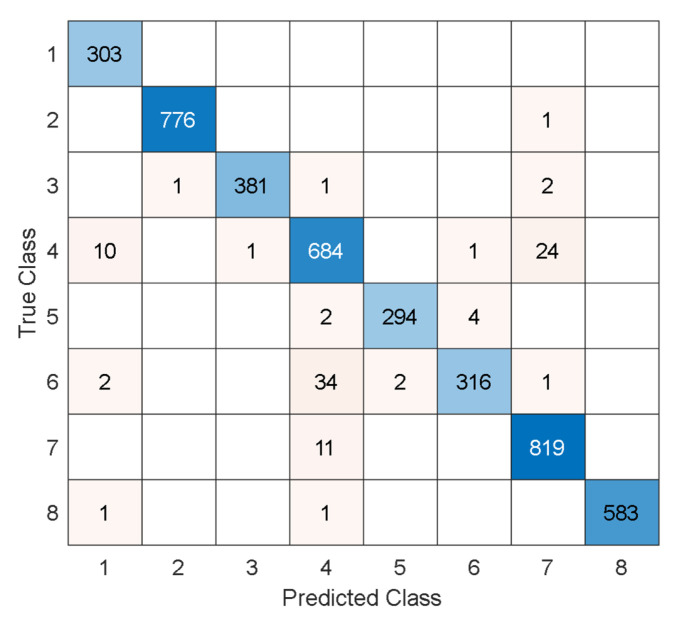
Test confusion matrix of the MACNeXt for the blood cell image dataset. The classes are given as follows. 1: Basophil, 2: Eosinophil, 3: Erythroblast, 4: Immature granulocyte, 5: Lymphocyte, 6: Monocyte, 7: Neutrophil, and 8: Platelet.

Also, the test results for the blood cell image dataset are computed, and the test confusion matrix is showcased in Figure 8.

Per Figure 8, the recommended model attained over 97% test results, and these results have been listed in Table 7.

Also, the comparative results for the blood cell image dataset are given in Table 8.

These results (see Table 8) openly demonstrated that the presented MACNeXt CNN has both high classification performances with fewer learnable parameters.

We also tested the recommended MACNeXt on two additional datasets: (i) the public DIBaS dataset and (ii) a blood-cell image dataset. The introduced MACNeXt illustrated high and stable performance on both of them. These results match the findings from our new real-world dataset and confirm that the model works well across different biomedical images. Overall, the experiments show that MACNeXt is a reliable and practical architecture that can outperform standard CNNs in a wide range of image-classification tasks.

### 4.2. Future Directions

The future directions are given below.

- Our proposed MACNeXt model will be evaluated on additional bacterial groups. These additional species are linked to the biocorrosion of medical implants.
- These bacteria affect material degradation and can influence long-term implant performance.
- The introduced MACNeXt can be adapted to these cases by training on corrosion-related colony images.
- We plan to collect corrosion-focused datasets from laboratory and clinical environments.
- The introduced MACNeXt architecture will be tested under both clinical and material-science settings to explore broader use cases.
- A Transformer-based version of the model will also be investigated to compare feature extraction, attention behavior, and scalability under the same systematic workflow.

## 5. Conclusions

This research presents a lightweight CNN named MACNeXt. The model has 4.4 million parameters and uses two activation functions and a multi-branch structure. The design improves feature extraction and keeps the model efficient.

The introduced MACNeXt achieved 90.97% accuracy, 89.63% precision, 88.64% recall, and 88.99% F1-score on our curated dataset with 24 species. The introduced MACNeXt model also achieved over 99% accuracy on the DIBaS dataset. In addition, MACNeXt attained 97.67% accuracy on the blood cell dataset. These results show strong performance and stable generalization across different biomedical images.

The compact structure offers benefits. Our presented MACNeXt uses low memory. It gives fast predictions. It works well on limited hardware. The overall design supports clear and reproducible decisions in clinical settings.

The MACNeXt provides a reliable and effective framework for bacterial and biomedical image classification. We plan to expand the species set, test the model in multiple centers, and design CNN and Transformer extensions to attain higher accuracy and reliability.

## Figures and Tables

**Table 1:** Number of samples for each bacterial species in the dataset.

Class No.	Bacterial Species	Number of Samples	Representative Image	Class No.	Bacterial Species	Number of Samples	Representative Image
1	*Acinetobacter baumannii*	535	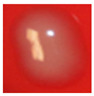	13	*Morganella morganii*	177	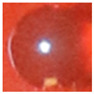
2	*Burkholderia cepacia*	272	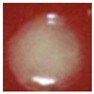	14	*Proteus mirabilis*	387	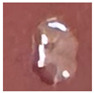
3	*Citrobacter freundii*	337	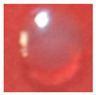	15	*Staphylococcus* *saprophyticus*	209	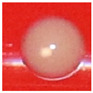
4	*Hemolytic* *Escherichia coli*	1860	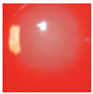	16	*Staphylococcus aureus*	1364	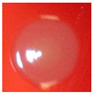
5	*Non-hemolytic Escherichia coli*	2562	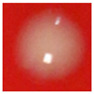	17	*Staphylococcus epidermidis*	2078	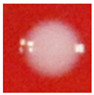
6	*Enterobacter* *aerogenes*	292	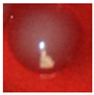	18	*Staphylococcus* *haemolyticus*	311	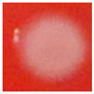
7	*Enterobacter cloacae*	208	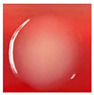	19	*Staphylococcus* *hominis*	527	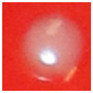
8	*Enterococcus gallinarum*	215	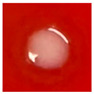	20	*Stenotrophomonas* *maltophilia*	306	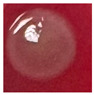
9	*Enterococcus faecalis*	2214	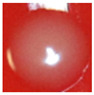	21	*Streptococcus* *pyogenes*	199	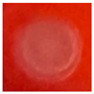
10	*Enterococcus faecium*	1013	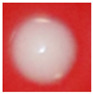	22	*Streptococcus* *agalactiae*	265	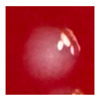
11	*Klebsiella oxytoca*	882	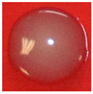	23	*Streptococcus* *anginosus*	256	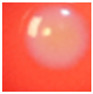
12	*Klebsiella pneumoniae*	1378	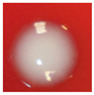	24	*Streptococcus mitis*	374	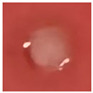

The dataset split was as follows: 64% training, 16% validation, and 20% testing for evaluation.

**Table 2:** Transition table of the presented MACNeXt. Herein ⨁: Addition layer.

**Layer**	Operation	Input	Output
Stem	$\left[\begin{array}{c} 3\times 3,\;48,BN,Stride:2\\ GC,3\times 3,\;96,G,BN,\;Stride:2 \end{array}\right]$	224 × 224 × 3	56 × 56 × 96
Main Stage 1	$\left[\begin{array}{c} \begin{array}{c} Concat\left(\left(GC,3\times 3,96,BN\right),\left(GC,1\times 1,96,BN\right)\right)\\ 1\times 1,\;192,G\\ GC,1\times 1,\;96,BN \end{array}\\ GC,1\times 1,\;96,G\;⨁\;GC,1\times 1,96,R \end{array}\right]\times 1$	56 × 56 × 96	56 × 56 × 96
Downsampling 1	$\left[GC,3\times 3,192,Stride:2\right]$	56 × 56 × 96	28 × 28 × 192
Main Stage 2	$\left[\begin{array}{c} \begin{array}{c} Concat\left(\left(GC,3\times 3,192,BN\right),\left(GC,1\times 1,192,BN\right)\right)\\ GC,1\times 1,\;384,G\\ 1\times 1,\;192,BN \end{array}\\ GC,1\times 1,\;192,G\;⨁\;GC,1\times 1,\;192,R \end{array}\right]\times 1$	28 × 28 × 192	28 × 28 × 192
Downsampling 2	$\left[GC,3\times 3,384,Stride:2\right]$	28 × 28 × 192	14 × 14 × 384
Main Stage 3	$\left[\begin{array}{c} \begin{array}{c} Concat\left(\left(GC,3\times 3,384,BN\right),\left(GC,1\times 1,384,BN\right)\right)\\ GC,1\times 1,\;768,G\\ 1\times 1,\;384,BN \end{array}\\ GC,1\times 1,\;384,G\;⨁\;1\times GC,1,\;384,R \end{array}\right]\times 3$	14 × 14 × 384	14 × 14 × 384
Downsampling 3	$\left[GC,3\times 3,768,Stride:2\right]$	14 × 14 × 384	7 × 7 × 768
Main Stage 4	$\left[\begin{array}{c} \begin{array}{c} Concat\left(\left(GC,3\times 3,768,BN\right),\left(GC,1\times 1,768,BN\right)\right)\\ GC,1\times 1,\;1536,G\\ 1\times 1,\;768,BN \end{array}\\ GC,1\times 1,\;768,G\;⨁\;GC,1\times 1,\;768,R \end{array}\right]\times 1$	7 × 7 × 768	7 × 7 × 768
Output	GAP Layer, Fully Connected Layer, Softmax Layer	7 × 7 × 768	Number of classes
Total Learnable Parameters	4.4 Million		

Table 2 showcases the operation types, kernel sizes, stride values, activation functions, and output dimensions for each layer of the proposed model. The notation R: [1, 1, 3, 1] and F: [96, 192, 384, 768] indicates the block repetition counts and corresponding filter numbers consecutively.

**Table 3:** Comprehensive listing of all model training parameters with detailed descriptions.

Parameters	Value
Split Ratio	Training: 64%, Validation: 16%, Test: 20%
Solver	SGDM
Epoch	30
Mini Batch Size	128
Initial Learning rate	0.01

**Table 4:** Test results of the recommended MACNeXt on the curated bacteria image dataset.

Metric	Results (%)
Accuracy	90.97
Precision	89.63
Recall	88.64
F1-Score	88.99

**Table 5:** Summary of recent studies on bacterial colony classification using machine learning and deep learning techniques, along with their reported performance metrics.

Author(s)	Aim/Number of Classes	Method	Performance (%)
Li et al. [6]	3-class bacterial colony detection (E. coli, Citrobacter, K. pneumoniae)	TFT-based lens-free imaging + ResNet	Accuracy = 97.3
Yang et al. [7]	Bacterial colony detection	Data augmentation via style transfer + Cascade Mask R-CNN/YOLOv8x	YOLOv8x = 76.7 mAP
Wu ve Gadsden [8]	33-class bacterial classification	DenseNet-121, ResNet-50, VGG16	Accuracy = 99.08
Makrai et al. [12]	Creation of a large-scale colony dataset containing 24 species	Bounding-box-based colony annotation	Dataset presentation
Talo [13]	33-class bacterial classification (DIBaS dataset)	Transfer learning + ResNet-50	Accuracy = 99.2
Akbar et al. [14]	Bacterial classification	Hybrid: ResNet-101 + SVM	Accuracy = 99.61
Gallardo-Garcia et al. [15]	Evaluation of mobile-friendly models (DIBaS dataset)	EfficientNet-Lite0, MobileNetV2	Accuracy = 97.38
Mai ve Ishibashi [16]	Development of a lightweight, low-parameter model (DIBaS dataset)	Depthwise Separable CNN	High accuracy (value not specified)
Zieliński et al. [17]	33-class bacterial classification (DIBaS dataset)	Convolutional Neural Network (CNN)	Accuracy = 97.24
Hallström et al. [18]	4-class bacterial classification (time-series images)	Video-based ResNet	Accuracy = 99.55 ± 0.25
Kang et al. [19]	Single-cell-level bacterial classification	Hyperspectral microscopy (HMI) + 1D-CNN	Accuracy = 90.0
Abd Elaziz et al. [20]	33-class bacterial classification (DIBaS dataset)	Hybrid: Fractional-order orthogonal descriptors + semantic features	Accuracy= 98.68
Our Method	33-class bacterial classification (DIBaS dataset)	Proposed MACNeXt model	Accuracy= 99.44 Precision= 99.43 Recall= 99.41 F1-Score= 99.41
Our Method	24-class clinical bacterial classification (Our collected dataset)	Proposed MACNeXt model	Accuracy= 90.97 Precision= 89.63 Recall= 88.64 F1-Score= 88.99

**Table 6:** Training and validation results of the introduced MACNeXt for the blood cell image dataset.

Metric	Results (%)
Training Accuracy	100%
Validation Accuracy	97.82%
Training Loss	9.71 × 10^−5^
Validation Loss	0.0804

**Table 7:** Test results of the recommended MACNeXt on the blood cell image dataset.

Metric	Results (%)
Accuracy	97.67
Precision	97.91
Recall	97.40
F1-Score	97.65

**Table 8:** Comparative results for blood cell image dataset [34,35].

Study	Method	Accuracy
[34]	VGG16	~96.0
[36]	Lightweight CNN	97.65
[35]	Deep CNN	96.10
[37]	Deep Neural Network	95.23
[38]	Custom CNN	97.60
[39]	Proposed model	97.67

## Data Availability

The data presented in this study are available on request from the corresponding author due to privacy restrictions and the use of private data.
